# A Novel Hierarchical Extreme Machine-Learning-Based Approach for Linear Attenuation Coefficient Forecasting

**DOI:** 10.3390/e25020253

**Published:** 2023-01-30

**Authors:** Giuseppe Varone, Cosimo Ieracitano, Aybike Özyüksel Çiftçioğlu, Tassadaq Hussain, Mandar Gogate, Kia Dashtipour, Bassam Naji Al-Tamimi, Hani Almoamari, Iskender Akkurt, Amir Hussain

**Affiliations:** 1 Department of Neuroscience and Imaging, University of Chieti Pescara, 66100 Chieti, Italy; 2 DICEAM, University Mediterranea of Reggio Calabria, Via Graziella, Feo di Vito, 89060 Reggio Calabria, Italy; 3 Department of Civil Engineering, Manisa Celal Bayar University, 45140 Manisa, Turkey; 4 School of Computing, Merchiston Campus, Edinburgh Napier University, Edinburgh EH10 5DT, UK; 5 School of Computing and Digital Technology, Birmingham City University, Birmingham B4 7XG, UK; 6 Faculty of Computer and Information Systems, Islamic University of Madinah, Medina 42351, Saudi Arabia; 7 Physics Department, Suleyman Demirel University, 32260 Isparta, Turkey

**Keywords:** hierarchical extreme machine learning, linear attenuation coefficient, XCOM

## Abstract

The development of reinforced polymer composite materials has had a significant influence on the challenging problem of shielding against high-energy photons, particularly X-rays and γ-rays in industrial and healthcare facilities. Heavy materials’ shielding characteristics hold a lot of potential for bolstering concrete chunks. The mass attenuation coefficient is the main physical factor that is utilized to measure the narrow beam γ-ray attenuation of various combinations of magnetite and mineral powders with concrete. Data-driven machine learning approaches can be investigated to assess the gamma-ray shielding behavior of composites as an alternative to theoretical calculations, which are often time- and resource-intensive during workbench testing. We developed a dataset using magnetite and seventeen mineral powder combinations at different densities and water/cement ratios, exposed to photon energy ranging from 1 to $100^{6}$ kiloelectronvolt (KeV). The National Institute of Standards and Technology (NIST) photon cross-section database and software methodology (XCOM) was used to compute the concrete’s γ-ray shielding characteristics (LAC). The XCOM-calculated LACs and seventeen mineral powders were exploited using a range of machine learning (ML) regressors. The goal was to investigate whether the available dataset and XCOM-simulated LAC can be replicated using ML techniques in a data-driven approach. The minimum absolute error (MAE), root mean square error (RMSE), and R2score were employed to assess the performance of our proposed ML models, specifically a support vector machine (SVM), 1d-convolutional neural network (CNN), multi-Layer perceptrons (MLP), linear regressor, decision tree, hierarchical extreme machine learning (HELM), extreme learning machine (ELM), and random forest networks. Comparative results showed that our proposed HELM architecture outperformed state-of-the-art SVM, decision tree, polynomial regressor, random forest, MLP, CNN, and conventional ELM models. Stepwise regression and correlation analysis were further used to evaluate the forecasting capability of ML techniques compared to the benchmark XCOM approach. According to the statistical analysis, the HELM model showed strong consistency between XCOM and predicted LAC values. Additionally, the HELM model performed better in terms of accuracy than the other models used in this study, yielding the highest R2score and the lowest MAE and RMSE.

## 1. Introduction

The use of X-ray and gamma beams in industry and medical imaging [1,2], the radiotherapy for the treatment of cancer [3], the gamma irradiation of plants for breeding mutations [4] and X-ray fluorescence (XRF) for elemental analysis [5] are examples of high-energy photon-based processes. Long-term exposure to high-energy photons, however, may be harmful to both users and the general public [6], with side effects including skin burn, nausea, vomiting, stomach discomfort, fever, diarrhea, hair loss, damage to the bone marrow, cancer, and even death [3]. Concrete is by far the most often used construction material. Reinforced concrete (RC) buildings, which are mostly constructed of cement, gravel, and water, have recently gained a great deal of attention thanks to their low cost, availability, and extensive applicability. Their gamma-ray and neutron attenuation characteristics, such as the linear attenuation coefficient (LAC, μ, cm${}^{2}$/g) mass attenuation coefficients (μ/ρ, cm${}^{2}$ g${}^{-1}$), effective atomic numbers ($Z_{eff}$), effective electron densities ($N_{eff}$), effective fast neutron removal cross-section (σ, cm${}^{-1}$), and effective mass removal cross-section (σR/ρ, cm${}^{2}$ g${}^{-1}$), are frequently determined in order to evaluate specific shielding characteristics. As the absorber’s ($Z_{eff}$) value grows, the linear attenuation coefficient for gamma ray shielding typically climbs due to high-$Z_{eff}$ components having stronger photoelectric and paired structure interactions. Hematite, geothite, limonite, magnetite, and barite are among the heavy metals and minerals used in concrete mixtures to boost photon resistance [7,8,9]. The literature contains both theoretical and empirical studies on the linear attenuation coefficients for various types of mineral powders and heavy metal mixtures at various photon energies. Magnetite (Fe${}_{3}$O${}_{4}$) is typically utilized in concrete mixtures as the primary heavy component. Together, concrete and magnetite create a thick (4.9–5.2 g/cm${}^{3}$), efficient shielding material for γ-rays [10,11]. The shielding property of magnetite may be accessed using the linear attenuation coefficient μ, commonly known as the possibility of radiation interfering with a substance [12]. It should be clearly stated that it is not always feasible for manufacturers of radiation-shielding concrete to evaluate a wide variety of mineral powders and/or their mixing ratios with heavy nanoparticles on a workbench in the lab. As a result, computational approaches such as Monte Carlo code [13,14,15] or the National Institute of Standards and Technology, the NIST photon cross-section database (XCOM) software are commonly used [8,9,16,17,18,19,20,21,22,23]. The XCOM database has been used in several academic articles to determine the (μ/ρ) rate for various elements [24], composites [25], compounds [26,27], concretes [28,29], glasses [30,31,32], polymers [33,34], rocks [31,35], construction materials [36], alloys [37], ceramics [38], and biological materials [39,40]. Calculating the right (μ/ρ) of various kinds of mixes and composite mineral powders requires a range of known material mix ratios. Over the past ten years, researchers have used computational modeling [41], parametric multi-variable regression modeling [42], and artificial intelligence [43] approaches to predict concrete samples radiation efficacy. Recent research has shown that gradient-based deep learning (DL) neural networks significantly outperform traditional machine learning (ML) approaches for a range of classification and regression applications [44,45,46]. However, these techniques require large amounts of labeled data in order to learn the mapping function and achieve consistent performance. Furthermore, a domain mismatch problem impairs the generalization performance of DL-based models. As a result, the performance of these models may considerably lower the system’s overall efficiency. The authors in [47] described a hierarchical structure of extreme learning machine (HELM) based on DL-based neural architectures, where features are retrieved and optimized in a multi-layer manner. In contrast to extreme learning machine (ELM), HELM uses a multilayer technique to extract data, keeping the advantages of DL neural models in approximate complex functions while maintaining strong classification/regression capabilities. Motivated by the promising results recently achieved in HELM-based models [48,49,50], this study aims at developing an LAC prediction framework based on HELM theory. Specifically, by leveraging HELM, a data-driven model for LAC prediction can be deployed with little training data and low processing capabilities.

The main contributions of the present paper are listed as follows: (i) Processing and analysis of linear attenuation coefficients (LAC) by XCOM; (ii) Development of a HELM-based method for predicting LAC in concrete mixes; (iii) Deployment of the proposed system in the industrial sector.

The paper is organized as follows: related works are shown in Section 2. In Section 3, the proposed HELM-based approach for LAC forecasting is presented together with the dataset on mineral powders and magnetite mixtures. The implementation of the developed ML prediction models is also thoroughly explored. Experiment results are reported in Section 4. Section 5 discusses and Section 6 concludes the paper.

## 2. Related Works

There are currently few studies on both the experimental simulation and AI techniques accessible in the subject of attenuation coefficient prediction using machine learning models, which is a relatively new area of study. For example, Gancel et al. (2009) [51] used Monte Carlo simulations and Artificial Neural Network (ANN) algorithms to forecast the mass attenuation coefficient of harzburgite material. Akkurt et al. (2010), developed a fuzzy logic model to forecast the shielding properties of concrete mixtures at different rates of powders and water [21]. Furthermore, Akkurt and colleagues compared XCOM outcomes with to the fuzzy logic findings. Başyigit et al. (2010) aim was to predict the compressive strength of barite aggregate concrete with artificial neural network and fuzzy logic methods [52]. Başyigit and colleagues asserted that these models are successfully founded fuzzy logic methods effective in estimating the compressive strengths of concretes containing barite aggregates. Gencel et al. (2013) improved a Mamdani-type fuzzy logic model to forecast the elasticity modulus and compressive strength of concretes with hematite [53]. It was concluded that the model gives consistent results with the values obtained via experimental simulation. Juncai et al. (2015) used the least squares-support vector machine (LS-SVM) to calculate the radiation shielding efficiency of different particles in concrete combinations [54]. In [54], the research aim was to determine the durability of radiation-shielding concrete. Furthermore, in [54], to identify the model’s ideal parameters, the parameter tuning was applied using the grid search approach. By contrasting the model’s results with the experimental data, the LS-SVM success was identified. Yadollahi et al. (2016) [55] used the Taguchi technique to establish the appropriate proportion of lead-slag aggregate in concrete mixtures, and an ANN was employed to assess concrete’s radiation-shielding capabilities. In Medhat et al. (2018) [56], the authors used the ANN technique to forecast the mass attenuation coefficient in building materials, glasses, and soil materials. The outcomes were compared with data collected using the XCOM program. Sayed et al. (2022) used (ANN) to predict the values of RCS for the different concrete mixtures [57]. The experimental findings and the forecast results from the developed ANN were highly congruent, and the produced models’ predictive power outperformed regression analysis. In Amin et al. (2022) ANN and gene expression programming (GEP) models were developed for predicting the radiation shielding capacity of concrete [58]. According to the statistical analysis, the generated models showed strong consistency between experimental and predicted findings. In contrast, the ANN model outperformed the GEP models in terms of accuracy, producing the greatest R and the lowest MAE and RMSE.

## 3. Materials and Methods

**Figure 1:**
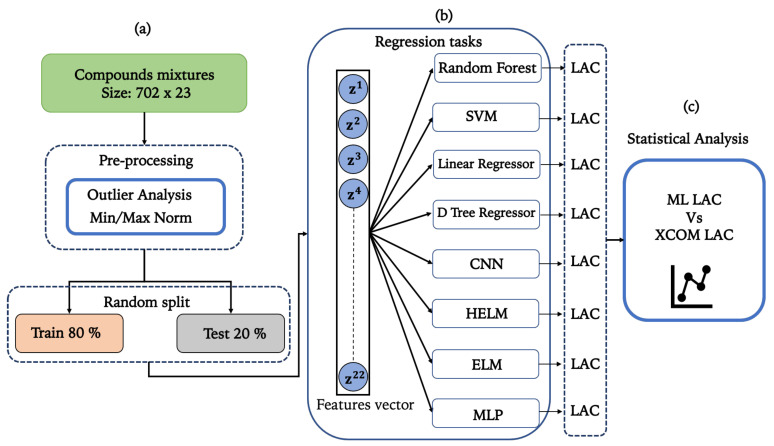
Overview of the study’s methodology. In order to create the dataset, samples of mineral powders, magnetite, gamma energies, and densities were first chosen. Then, Panel (**a**) shows the preprocessing stage, which included out-layer removal and min-max normalization. The preprocessed data were then split into train (80%) and test (20%) groups at random. In Panel (**b**), the features vector is emphasized. Using the train dataset and the 10 k-fold cross-validation procedure, we trained our ML regressor models. To quantify the performance of the proposed ML regressors in Panel (**c**), we calculated regression and statistical analysis comparing the ML results to the XCOM simulated LAC.

Figure 1 reports the flowchart of the proposed procedure. Each processing block is detailed in the following subsections.

### 3.1. Materials

Portland cement (PC) of type I, CEM I 42.5 was combined with 17 mineral powders at five different concentration percentages. Heavy magnetite was added to the final mixing at five different concentrations to increase the concrete’s shielding properties. Table 1 displays the powder particles of the magnetite/mineral powders mixture combinations at different water and density ratios.

### 3.2. Mixing Procedure

The properties of the concrete shields were designed using heavy material mixes and mineral powders at various water/density ratios. To achieve the desired degree of strength, all heavy-weight mixtures have to maintain a constant water-to-density ratio; thus, we used the following range of water $H_{2}O$ = {0.0781, 0.07512,0.0717, 0.0687, 0.0659} g/cm${}^{3}$. Five different densities were used to determine the unit weights of the concrete mixtures *d* = {2.44, 2.55, 2.67, 2.78, 2.9} g/cm${}^{3}$. The mixtures were prepared by combining Portland cement with other conventional heavy-weight aggregates that included magnetite powder at five different percentages, $d^{\prime }$ = {0, 0.25, 0.5, 0.75, 1} g/cm${}^{3}$. Finally, we use a range of γ with 108 different energies from 1 to $100^{6}$ (KeV) photon energies.

### 3.3. Chemical and Physical Characteristics

In order to conduct the radiation attenuation measurements, the samples were prepared and processed. Concrete is a composite material whose mechanical, physical, and chemical characteristics are determined by the mix design and manufacturing method. Mix design includes deciding on the kind and amounts of aggregates, cement, water, chemical or mineral powders, and their mixtures, whereas manufacturing procedures entail batching, mixing, transporting, putting, compacting, and curing new concrete in a semi-controlled environment. In this study, magnetite is the main heavy-weight aggregate. The specimens must be of the material in the form of a homogeneous powder of the substance for chemical composition measurement; therefore, a portion of each sample was dried and ground, yielding an ultimate powder specimen weight of 100 gr. On the other hand, in order to help with the estimation of their density during tests, the specimens used to establish the mass attenuation coefficient must be solid and generally in a regular form. The specimens must be reasonably thin in order to provide the proper geometry for the detection of transmitted gamma rays through the specimen. Their dimensions must match those of the detector and the gamma-ray source in order to suppress radiation effectively. The detail of the mineral powders densities are summarized in Table 1, the mixtures process and ratio in Section 3.2.

### 3.4. XCOM Simulation

The total mass attenuation coefficient at various shielding mineral powders and heavy aggregate mixtures and different energies may be determined using the relevant database XCOM. Additionally, it has the capacity to calculate partial derivatives (photoelectric, Compton scattering, and pair production interactions). The mass attenuation coefficient of various types of mineral powders might theoretically be calculated using the sample’s compound weight fractions as input into the XCOM algorithm. We used the gamma transmission technique, which is based on the absorption of gamma rays through material, to compute the mass attenuation coefficients experimentally. The mass attenuation coefficients of magnetite and mineral powders were simulated using a range from 1 to $100^{6}$ (KeV) photon energies of commonly used radionuclides. Here, we used mineral powders like (CaO, MgO, NaO, K${}_{2}$0, Fe${}_{2}$O${}_{3}$, P${}_{2}$O${}_{5}$, CO${}_{2}$, SiO${}_{2}$, Al${}_{2}$O${}_{3}$, SO${}_{2}$, TiO${}_{2}$, BaO, Cr${}_{2}$O${}_{3}$, MnO, Na${}_{2}$O, SO${}_{3}$, V${}_{2}$O${}_{5}$, and ZnO) and XCOM software [59] to simulate the LAC characteristics of mixtures. For the forecasting analysis, we used the XCOM-simulated LACs as the ground truth. The linear attenuation coefficients were estimated using Equation (1) after these coefficients had been determined based on the elemental percentages of these mixtures as listed in Table 1.

(1) $$I=I_{0}e^{-\mu_{m}\rho x}.$$

where the μ for any mineral powder or the mixture of elements can be calculated in terms of the weight fraction ($\omega_{i}$) by using the following equation:

(2) $$\mu_{m}=\sum W_{i}{\frac{\mu }{\rho }}_{i}.$$

where $W_{i}$ and $\mu_{m}$ are the weight fraction and the mass attenuation in (cm${}^{2}$/g) for *i*th constituent element. Here, the $W_{i}$ weight fraction of the *i*th constituent element is given by:

(3) $$W={\frac{n_{i}A_{i}}{\sum_{j}^{}n_{j}A_{j}}}_{i}.$$

where $A_{i}$ is the atomic weight and $n_{j}$ is the number of formula units.

### 3.5. Database Organization

We designed a dataset $A_{\left(i,j\right)}\in {ℜ}^{\left(n\times m\right)}$, where m and n are, respectively, 702 and 23. Let *A* = $\{T_{1}$, $T_{2}$, ⋯, $T_{m}\}$ be the dataset structure and ∀$T_{i}$⊂$A^{\prime }$, we have $A_{\left(i,1\right)}^{\prime }$ = $T^{\prime }$. Here, $T^{\prime }$ represents a column in $A^{\prime }$. $T_{\left(1,\cdots ,n\right)}$ contains the following labels (Energy (keV), magnetite%, density, CaO, MgO, NaO, K${}_{2}$0, Fe${}_{2}$O${}_{3}$, P${}_{2}$O${}_{5}$, CO${}_{2}$, SiO${}_{2}$, Al${}_{2}$O${}_{3}$, SO${}_{2}$, TiO${}_{2}$, BaO, Cr${}_{2}$O${}_{3}$, MnO, Na${}_{2}$O, SO${}_{3}$, V${}_{2}$O${}_{5}$, ZnO, and finally the LAC). Further details are reported in Table 1.

### 3.6. Data Preprocessing

The suggested step organizes data so that it may be processed properly by the following modules. Data normalization and outliers analysis are two sub-stages.

### 3.7. Outliers Analysis

Before data normalization, the concrete dataset is screened to remove outliers. Outliers are known to cause a loss of accuracy in commonly used forecasting models by interfering with the learning process. We used the median absolute deviation estimator (MADE) to identify outliers. MADE is a robust scale estimator which is defined as follows:

(4) $$MADE=Q*med(z_{\left(i,j\right)}-|med\left(z_{\left(i,j\right)}\right)|)$$

Here, med is the median operator, whereas $z_{\left(i,j\right)}$ is the sample of the characteristic $z_{f}$, and *Q* = 1.4826 [60] is a multiplicative constant commonly used when data is assumed to be normal. The MADE provides an output matrix as defined in Equation (4). In this paper, $z_{\left(i,j\right)}$ is considered an outlier if

(5) $$z_{f,i}=>Q*MADE$$

Finally, after removing the out-layers, the dataset size of our dataset A changed from 15.466 to 14.758 items.

### 3.8. Data Normalization

In order to prevent false predictions caused by the high magnitude of the energy radiation used, the dataset *A* is regularized using the min-max normalization approach. Using the min-max normalization approach, the numerical feature values $z_{i,j}$ were translated into the numeric range [0–1] as follows:

(6) $$z_{\hat{f},i}=\frac{z_{f,i}-min\left(z_{f}\right)}{max\left(z_{f}\right)-min\left(z_{f}\right)}$$

where $max(z_{f})$ and $min(z_{f})$ denote the maximum and minimum values of the *f*th (numeric) feature zf, and $z_{\left(f,i\right)}$ denotes the normalized feature value spanning from 0 to 1.

### 3.9. ML Input Data Organization

Let X ∈$R^{\left(n\times m\right)}$ and Y ∈$R^{\left(n\times 1\right)}$ be a regular matrix with X${}^{\left(n\times m\right)}$. Where X represents the n×m raw and columns of Table 1 and Y the XCOM forecasted LAC. The dataset A = XY, is partitioned into training $\{A_{Train}$; $y_{Train}\}$, and test set $\{A_{Test}$; $y_{Test}\}$ in the ratio of 80/20 using the train/test split function from sklearn library in python. Here, $A_{Train}$⊂ X represents 80% of the prepossessed data. Given $A_{Train}$⊂ A, a 10 k-fold cross-validation procedure is applied on $A_{Train}$. Using this approach ten training $\{A_{Train}^{\prime }$; $y_{Train}^{\prime }\}$, and test $\{A_{Test}^{\prime }$; $y_{Test}^{\prime }\}$ matrices are built. The input of each forecast model is represented as a fully observed feature vector set Y = $\{\left(X_{\left(i,1\cdots ,23\right)},y_{i}\right\}$, with the corresponding label $y_{Train}^{\prime }\left(i\right)$. So, taking into account x’ = $\left\{A_{Train}^{\prime }\right\}$, a collection of mineral powders particles and y $\left\{y_{Train}^{\prime }\right\}$, LAC, the input of the supervised models are produced.

### 3.10. Conventional Shallow Machine Learning (ML) Classifiers

The concrete acts differently after being mixed with mineral powder particles and heavy components. As a result, various RSC aggregates or combinations may work differently due to their properties. Thus, it is crucial to take into account the radiation properties of mineral powder particle mixtures while developing the concrete radiation approach. These characteristics include the amount of water in the mineral powders particle mixtures, the amount of mineral powders particles, the amount of additives, and the grading of the heavy aggregate. We propose to employ ML to predict the RS strength. The implementation of this approach involved the following six steps.

- The dataset was created using an engineering design approach to estimate the amount and proportion of each component. The shielding properties of radioactive concrete and mineral powders particle mixtures were estimated using XCOM.
- To eliminate outliers and normalize the data, the mineral powders particles, magnetite, water, and radiation samples were fed into a preprocessing pipeline.
- The sample data were divided into training and test sets and fed into ML predictors. A grid search approach was used to improve the kernel parameters, penalty factor, and ML model parameters.
- Nonlinear relationships between concrete radiation strength and concrete mixes were found to be effectively captured by the ML models.
- The trained ML model weights were used to calculate concrete radiation strength, by effectively generalizing from unmonitored data to unseen test data.
- The predictability of ML models was assessed by contrasting forecasted samples with empirically calculated data.

In this work, the ability to predict LAC values of eight well-known machine learning approaches was investigated. One of the main objectives of this work is to evaluate accurate shallow models for forecasting radiation shielding coefficients in order to avoid utilizing resource-intensive algorithms to identify particular gamma radiation intensity. To predict LAC values, we used the following machine learning models: hierarchical extreme machine learning, extreme machine learning, CNN, MLP, SVM regressor, linear regressor, decision tree regressor, random forest regressor, and SVM regressor. The SVM regressor, linear regressor, decision tree, and random forest regressors have all been implemented using the Scikit-learn package [61]. We trained, adjusted, and tested each classifier individually using the dataset depicted in Section 3.2. Training and test sets are typically separated into ML datasets. Our dataset was randomly divided into training groups (80%) and test groups (20%) using the Scikit-Learn tool. The train/test dataset that was utilized in this analysis was too small to be further separated. The Scikit-learn package’s 10-fold cross-validation dataset partition approach was used in our analysis. This allowed us to calculate the classifier’s performance average across its 10 training and testing iterations. Cross-validation is a useful technique for preventing the rising randomness and over-fitting that might happen when there are not enough samples. Both the model optimization of feature selection and the combined hyper-parameter searches apply 10-fold cross-validation. Section 3.15 provides detailed information on parameter tuning. One Intel Core i7 quad-core CPU running MacOS BigSur at 2.2 GHz and 16 GB of RAM is used for all the experiments.

### 3.11. Extreme Machine Learning

In contrast to conventional machine learning techniques, Huang et al. [62] developed a powerful and straightforward ELM framework to manage improved generalization at a quick learning speed while avoiding the restrictions of the BP algorithms. The parameters do not need to be changed by BP because the ELM is a single layer feed-forward neural network (SLFN). The extended SLFNs hidden layer settings, such as input weights and biases, can be randomly initialized in order to learn *L* various observations. The ELM’s output can be expressed as in Equation  (7)

(7) $${f(y}_{i})=\;\sum_{l=1}^{L}\beta_{l}\;G\left(w_{l}\cdot y_{i}\;+\;b_{l}\right)\;$$

where $y_{i}={\left[y_{i1\;},\;\;y_{i2}\;,\;\cdots ,\;y_{iM}\right]}^{T}\in R^{M}$ is the vector of input data, $G\left(.\right)$ is the activation function, $w_{l}={\left[w_{l1\;},\;\;w_{l2}\;,\;\cdots ,\;w_{lM}\right]}^{T}\in \;R^{M}$ is a vector of input weight,$\;b_{l}$ is the bias of the *l*-th hidden node, $\beta_{l}=\;{\left[\beta_{l1\;},\;\;\beta_{l2}\;,\;\cdots ,\;\beta_{lN}\right]}^{T}\in R^{N}$, where *L* is the total number of hidden neurons, and is the output weight vector. The aforementioned relationship can compactly be written as

(8) $${f(y}_{i})=\;\sum_{l=1}^{L}\beta_{l}\;h_{l}\left(y\right)\;=h\left(y\right)\;B$$

where$\;h\left(y\right)$ is the output weight matrix, and B is the hidden layer feature mapping.

The B can be computed as

(9) $$B=H^{+}X$$

where $H^{+}$ is the Moore–Penrose (MP) pseudoinverse of the hidden layer matrix (H) which can be determined as follows:

(10) $$H=\underset{\Phi }{\underbrace{\left[\begin{array}{ccc} g(w_{i}x_{j}+b1) & \cdots & g(w_{\hat{n}}x_{1}+b_{\hat{n}})\\ \vdots & \vdots \\ g(w_{i}x_{n}+b1) & \cdots & g(w_{\hat{n}}x_{n}+b_{\hat{n}}) \end{array}\right]}}\underset{\Theta }{\underbrace{\left[\begin{array}{c} \Theta_{1}^{T}\\ \vdots \\ \Theta_{\hat{n}}^{T} \end{array}\right]}}\underset{T}{\underbrace{\left[\begin{array}{c} T_{1}^{T}\\ \vdots \\ T_{n}^{T} \end{array}\right]}}$$

ELM selects hidden layer parameters $\omega_{i}$ and $b_{i}$ randomly, and finally computes the output layer parameter $\Theta_{i}$ using Moore–-Penrose generalized inverse.

(11) $$\Theta =H^{T}{\left(HHT\right)}^{-1}T$$

Feedforward neural networks were the basis for the first development of ELM [62]. Therefore, a range of feature mappings (hidden-layer output functions), such as kernels and random hidden nodes, may be implemented using ELM. Strong classification and regression capabilities are offered by such kernel-based ELM.

### 3.12. Hierarchical Extreme Machine Learning (HELM)

An H-ELM framework for MLPs was proposed by Tang et al. [63]. Unsupervised hierarchical feature representation and supervised feature classification are the two distinct components of the H-ELM training architecture. The ELM-Based Sparse Autoencoder is used in the first stage to extract the input data’s multilayer sparse features. While for the latter, the original ELM algorithm is employed for the decision-making process. Algorithm 1 displays the pseudo-code for implementing an ELM-based sparse autoencoder.
**Algorithm 1** HELM Framework.1:**Input:** The matrix of the practice data X: ($x_{i}$)|$x_{i}$∈ Rd, *i* = 1, …, N;2:Function of a hidden node output: G($a_{i}$, $b_{i}$, x);3:The number of hidden nodes: l;4:Size of iterations;5:**Output:** Hidden node matrix V: ($a_{i}$, $b_{i}$), *i* = 1, …, l; β${}_{i}$;6:Compute the H hidden layer output matrix;7:Calculate the matrix $H^{T}H$;8:Determine the constant γ of the smooth convex function ∇, whose gradient relies on the largest eigenvalue;9:$y_{1}\overset{}{\leftarrow }\beta 0$∈$R^{n}$ and $t_{1}\overset{}{\leftarrow }1$;10:**for** (i = 0; i < x; i++) **do**11:    Calculate β${}_{k}$ using Equations (12) and (13):
(12)$$\beta_{k}\overset{}{\leftarrow }p_{L}\left(y_{k}\right)\overset{}{\leftarrow }T_{\alpha }\left(y_{k}-2\frac{1}{\gamma }H^{T}H+2\frac{1}{\gamma }H^{T}X\right)$$
(13)$$T_{\alpha }\left(x\right)\overset{}{\leftarrow }\left(|\beta |-\alpha +sng\left(\beta \right),\alpha \right)\overset{}{\leftarrow }\frac{\lambda }{\gamma }$$12:    Calculate $t_{\left(k+1\right)}$ using Equation (14):
(14)$$t_{\left(k+1\right)}\overset{}{\leftarrow }\left(\frac{1+\sqrt{1+4t_{k}^{2}}}{2}\right)$$13:    Calculate $y_{\left(k+1\right)}$ using Equation (15)
(15)$$y_{\left(k+1\right)}\overset{}{\leftarrow }\beta_{k}+\left(\frac{t_{k}-1}{t_{k}+1}\left(\beta_{k}-\beta_{\left(k-1\right)}\right)\right)$$14:**end for**15:**Return** $\beta_{k}$

In order to make use of hidden information inside training samples, the input raw data for each forward feature representation layer needs to first be converted into an ELM random feature space. The ELM-Based sparse autoencoder technique is then used to do multilayer unsupervised learning in order to finally obtain the high-level sparse features. Each hidden layer’s output may be modeled mathematically as follows:

(16) $$O_{i}=G\left(O_{\left(i-1\right)}\times \beta_{\left(i-1\right)}\right)$$

where $O_{i}$ represents the output of the ith layer, $O_{i}$ − 1 represents the output of the (i−1)th layer, G(·) stands for the hidden layers’ activation function, and β stands for the hidden layer’s (i−1)th output weights. Each hidden layer of the H-ELM system is a discrete module that serves as a feature extractor. The weights or parameters of the current hidden layer are fixed and do not require adjustment after the feature of the previously hidden layer has been retrieved. The kth layer’s outputs, $O_{k}$, are regarded as the high-level features that were retrieved from the input data using multilayer unsupervised feature learning. They are randomly disrupted before being used as the inputs of the supervised ELM to obtain the overall network’s classification results. Algorithm 2 displays the total training H-ELM framework algorithm.
**Algorithm 2** ELM-Autoencoder Overview.1:**Input:** The training dataset matrix X: $\left(x_{i}\right)|x_{i}\in$$R^{n}$, i = 1, …, N; The number of hidden layer for sparse autoencoder: m;2:**Output:** Output weight vector of each layer: β${}_{i}$;3:Randomly generate hidden node matrix for original ELM $V_{\left(m+1\right)}$,4:$O_{0}\overset{}{\leftarrow }X;$5:**for** (i = 0; i < x; i++) **do**6:    Calculate hidden weight vector β${}_{i}$ by Algorithm 1 using $O_{i-1}$ and $V_{i}$ as its parameters  7:    $O_{i}$←$O_{i-1}\times \beta$${}_{i}$8:**end for**9:Compute output weight β m + 1 by Equation (8) using $O_{m}$ and $V_{m}$ as its parameters;10:**Return** $\beta_{i},i=1,\ldots ,\left(m+1\right)$.

**Figure 2:**
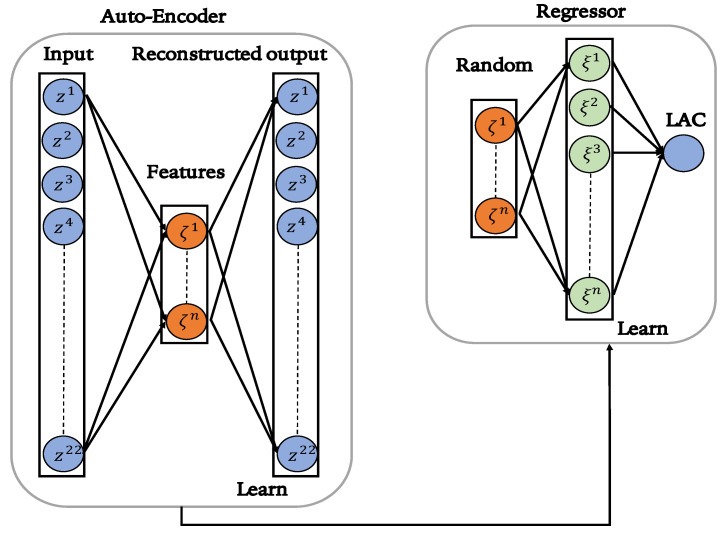
Flowchart of the proposed HELM architecture.

Figure 2 shows the unsupervised and supervised stages of The proposed HELM architecture.

### 3.13. Multi-Layer Perceptron

The most well-known feed-forward neural network is the multi-layer perceptron (MLP), which generally consists of an input layer, one or more hidden layers, and an output layer. It is trained to oversee learning processes using the popular backpropagation technique, as described in [64]. In recent years, concrete shielding propriety prediction in nuclear engineering has seen extensive usage of the ANN approach [65,66]. This paper suggests a methodology for LAC forecasting based on MLPs. The MLP design under consideration has an input layer with 22 neurons, a hidden layer with 10 neurons, and an output layer with 1 neuron. In the last layer, we use a softmax function as it is effective for forecasting.

### 3.14. Proposed 1d-Convolutional Neural Network (CNN)

A convolutional neural network’s architecture typically consists of two key elements: (i) A feature extractor that automatically pulls out a section of data from raw inputs. This technique convolves the input with a sliding kernel, an activation function, and pooling layers one or more times. (ii) In the last step, regression tasks are carried out utilizing a fully connected multi-layer neural network. If we let a 2d input map to be represented by *X*${}_{i}$∈ R${}^{\left(x_{i},y_{\left(1\cdots n\right)}\right)}$ and the weights of weight matrices (known as kernels) be represented by *K*${}_{i}$∈ R${}^{\left(x_{i},y_{\left(1\cdots n\right)}\right)}$, respectively (or filters), the convolution operation can be expressed as:

(17) $$Y_{i}=\sum_{i=1}^{n}X_{i}K_{i}$$

The maximum size of a data chunk that the network can process is n × F, where F is the total number of features in each validation set and n is the total number of columns in the dataset. Here, n and F are set to n = 22 and F = 211. After multiple trial runs, example filter size (k1 × k2) and number (K) are determined empirically. In this study, we use K = 32 learnable filters with a 1 × 11 size. Here *j*th filter convolves inside the X${}_{i}$ local region (also known as the receptive field), and then, using the same set of weights, slides over the whole input map with a stride (s) of s. With a stride of 1, the input data for each filter is convolved to create 32 feature maps with the same input size (211×22). Using the formula above, it is possible to obtain the output size as follows:

(18) $$y1=\frac{a-k_{1}+2p}{s}+1=\frac{22-1+2x0}{1}+1=22;$$

(19) $$y1=\frac{a-k_{2}+2p}{s}+1=\frac{211-11+2x0}{1}=201;$$

The 1d-CNN has W${}_{conv}$ = K × kernel size = 32 × 1 × 22 = 704 weights and $B_{conv}$ = 32 biases, resulting in a total of 704 + 32 = 736 learnable parameters. ReLu and MaxPool come after the convolutional layer, which shrinks the size of the feature maps from 201 × 22 to 191 × 22 when using a stride s=1. The features are extracted by following the equation below:

(20) $$y1=\frac{y-k}{s}+1=\frac{22-1}{1}+1=22;$$

(21) $$y2=\frac{y-k}{s}+1=\frac{201-11}{1}+1=191;$$

A fully connected (FC) layer with 22 hidden units receives flattened characteristics that are extracted at this stage. The number of hidden units (N), the quantity of filters (K), and the size of the output of the preceding convolutional layer (y1 × y2) all affect how many weights are used in the FC. The total weights are W${}_{conv}$− FC = y${}_{1}$× y${}_{2}$× K × FC = 4 × 192 × 32 × 22 = 540.672. The total number of parameters in this situation is 540.672 + 22(biases) = 540.694. Following recent research that has reported higher generalization performance in the learning process of CNN architectures, we utilize Rectified Linear Units (RELU f(x) = max(0, x)) as a nonlinear transfer function in the convolutional activation layer in this study [67]. Additionally we use the sigmoid (f(x) = $\frac{1}{\left(1+e^{-x}\right)}$) transfer function in the output layer. In order to reduce the size of the input feature maps ($Y_{j}$), the pooling layer of the CNN architecture uses the maximum number of nearby components selected using a filter of size $k_{1}$×$k_{2}$. According to Equations (20) and (21), the filter may scan the features map with stride, and generates a sub-sampled representation of $Y_{j}$ that is $y_{1}$×$y_{2}$. The CNN architecture’s learning parameters are adjusted in accordance with [68]. With a mini-batch size of 11, the stochastic gradient descent approach is used to train the convolutional network. This method uses a mini-batch learning strategy. We may refer to mini-batch as a sub-sample $x_{\left(1,\cdots ,n\right)}$ that is processed here at each iteration, taking into account that $x_{\left(1,\cdots ,k\right)}$ is the sample size of the LAC dataset used as input to the CNN. The weight-decay parameter is set at λ = 0.00001 and the learning rate parameter is fixed at α = 0.01. Since problems such as gradient vanishing or gradient explosion can still result in errors during training if weights are set too high or too low, determining the right range of random values can be difficult.

### 3.15. Parameter Tuning for Training

Any machine learning regression’s estimation accuracy is highly dependent on its customized parameters. These parameters are user-defined inputs that are optimized or tweaked depending on prior experiences. We used a GridSearchCV approach to optimize the hyper-parameters and identify the set of hyper-parameters that produces the best accurate results. Grid Search employs several combinations of the algorithms’ chosen hyperparameters, computes the model with each combination using cross-validation, and then displays the most effective combination. A dictionary with the names of hyperparameters as keys and a list of those parameters’ values is a hyperparameter grid. Using the hyperparameter grid, the GridSearchCV calculates the training accuracy and prediction scores for our models and displays all results for various combinations. The mean absolute error is used as a function to assess the quality of any split during the training of DT regression. The highest point in the tree’s depth corresponds to the level where the leaves are finest when the nodes divide. Because the tree contains more splits as it becomes deeper, a more overfitted model will be generated. This model will be suitable for the training data, but it will not generalize well to the test set [69]. The maximum depth is set at 10 during the training phase for all three variables in accordance with the highest R2scores. The number of iterations for training ML regression models is adjusted to 1000 since it results in a more satisfactory model error and training duration. The amount of model variance gained decreases with the number of iterations used [70]. The C parameter aims to impose a penalty for each misclassified data element during the training of SVM regression. A larger margin occurs at the SVM network and the penalty is minimal when it is configured with a lower value. The decision boundary will have a narrower margin if it is adjusted with a bigger value, which results in a higher penalty for misclassifications [71]. In our trials, the C parameter had no discernible impact on training accuracy, but higher values slowed down the processes; therefore, it was set to 1.0. Due to its higher accuracy ratings compared to other kernel types, the RBF is utilized as a kernel function. A gamma parameter must be adjusted once the RBF kernel function has been utilized. This parameter specifies the range of an SVM network training point’s effect. The more training points that are gathered together, the greater the gamma parameter’s value. The solver hyper-parameters and regularization parameter C are needed for the training of the linear regressor. With a regularization value C = 1.0, we corrected the lbfgs kernel hyper-parameter of the linear regressor. The maximum number of iterations, max_iter, and the parameter class_weight were both set to “balanced”. The next subsection is a better place to cover the various CNN, MLP, HEML, and EML models.

## 4. Experimental Results

The effectiveness of the learning model can be assessed using a variety of statistical approaches, depending on the discrepancy between the actual value and the anticipated value. The efficiency of regressor models was assessed in this study using the correlation coefficient (R2score), root mean square error (RMSE), and mean absolute error (MAE). The mean absolute error: mean absolute error determines the absolute difference between the expected value and the actual value as stated in Equation (22).

(22) $$MAE=\frac{1}{N}\sum_{i=1}^{N}\left|y_{i}-y_{i}^{pred}\right|$$

where *N* is the total number of samples, $t_{i}$ is the observed value of experimental samples, and $td_{i}$ is the forecast value. The MAE nearing “0” indicates that the model is effectively accurate. The root mean squared error is described as the standard deviation of variations between the predicted value and the actual value (23).

(23) $$RMSE=\sqrt{\frac{1}{N}\sum_{t=1}^{N}{\left|y_{i}-y_{i}^{pre}\right|}^{2}}$$

RMSE values close to “0” also denote good performance on the part of the model. The correlation between goals and outcomes is represented by the coefficient R2score [72]. In essence, a larger R2score (ranged from 0 to 1) shows better accuracy of the model, as opposed to lower RMSE and MAE [73]. We compute R2score as follows:

(24) $$R2score=1-\frac{\sum_{i=1}^{N}\left(y_{i}-\hat{y}\right)\left(y_{i}^{pre}-y^{\hat{p}re}\right)}{\sqrt{\sum_{i=1}^{N}{\left(y_{i}-\hat{y}\right)}^{2}\sum_{i=1}^{N}{\left(y^{pre}-{\hat{y}}^{pre}\right)}^{2}}}$$

where, $\hat{y}$ is the average of predicted values.

The correlation coefficient R2score is used to assess linear dependence between the actual and predicted values. When the R2score is close to zero, there is no evidence that the actual value is connected to the projected value; yet, when the R2score is close to one, the real value and projected value are almost identical. However, the R2score value does not significantly change when $y_{i}^{pre}$ is multiplied by a constant, hence it is insufficient to judge the accuracy of a model. RMSE and MAE are additional measures to confirm the goodness-of-fit of machine learning models. A decrease in error between actual and predicted values, which is often brought on by a higher R2score and a lower RMSE and MAE value, is a common indicator of a model’s accuracy. Table 2 provides a comparison of the regressor’s performance metrics. Table 2 provides normalized 0 to 1 values of MAE, RMSE, and R2score with mean ± standard deviation.

**Figure 3:**
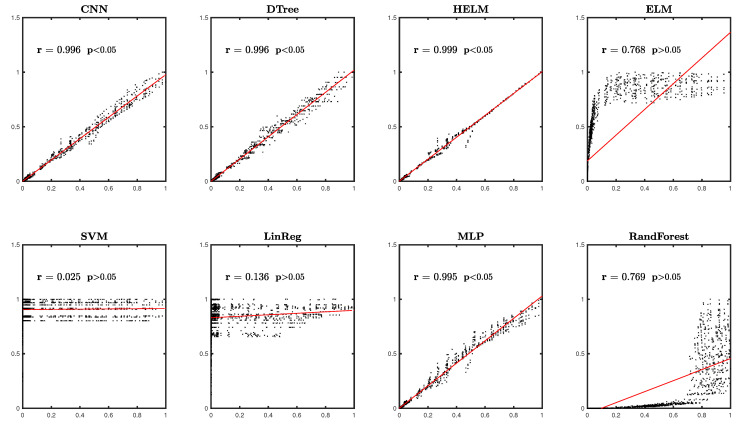
Regression plots and correlation analysis on LAC data predicted by the proposed ML models.

To evaluate how well our machine learning models worked, we used correlation and linear regression. Figure 3 illustrates how we assessed the performance of ML models by contrasting differences between the LAC predicted by our ML model and that simulated by XCOM.

**Figure 4:**
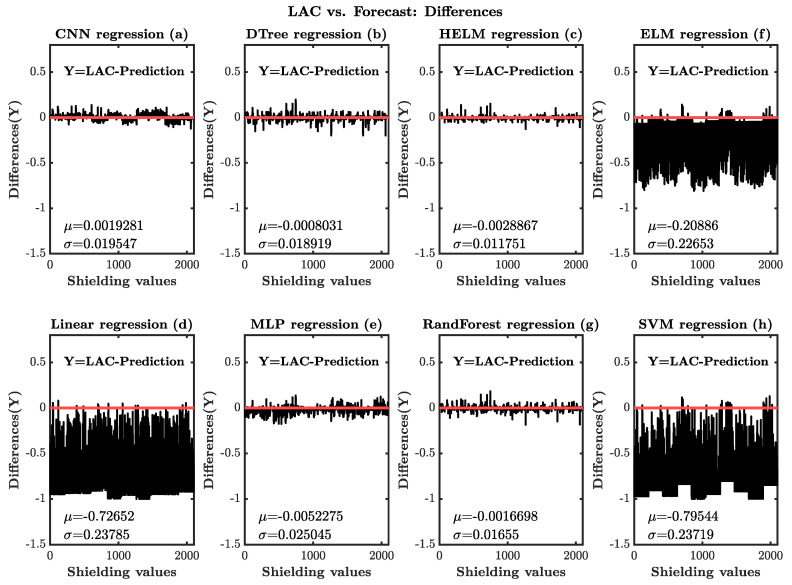
The figure presents the linear difference between input XCOM LAC and the LAC forecasted by the developed ML regressor models. Here x-axis represents the number of samples in the experiment, and y-axis represents the differences. Each panel also reports the mean and standard deviation.

Furthermore, we implemented a linear subtraction, *Y*=$XCOM_{LAC}$-$ML_{LAC}$, to access differences between the LAC predicted by our ML models and the LAC simulated by XCOM. Figure 4 illustrates the difference between predicted values and the actual data (XCOM).

We used the R2score, RMSE, and MAE measures to assess the prediction error rates and model performance during a forecast assignment. The performance of the algorithms is detailed in Table 2. Using a k-fold validation method, we found that the normalized MAE of {0.441 ± 0.356, 0.457 ± 0.314, 0.328 ± 0.301, 0.553 ± 0.303, 0.359 ± 0.374, 0.360 ± 0.304, 0.442 ± 0.356, 0.574 ± 0.387} keV/μm, respectively, for VM, decision tree, polynomial regression, random forest, MLP, CNN, ELM and HELM. Where the average of the absolute differences between the dataset’s actual and projected values over each iteration is represented by the mean absolute error. The RMSE was calculated in terms of mean across each iteration rate and quantifies the standard deviation of residuals. Then, for both, the standard deviation of results across iterations was computed. The coefficient of determination, commonly known as R-squared, indicates how much of the variance in the dependent variable the linear regression model can explain. The score is scale-free; thus, regardless of how big or little the numbers are, it will always be less than one. The correlation score, here R2score was {0.286 ± 0.315, 0.437 ± 0.293, 0.369 ± 0.321, 0.439 ± 0.305, 0.667 ± 0.277, 0.753 ± 0.292, 0.516 ± 0.296, 0.530 ± 0.334}, respectively, for CNN, DTree, HELM, ELM, SVM, LinReg, MLP, RandForest.

## 5. Discussion

Comprehensive investigations on the mass attenuation coefficient and radiation shielding of concrete made with different aggregates and construction supplies are available in the literature. In silico simulation is a reliable substitute for damaging, expensive, and time-consuming heating and chemical processes for concrete sample composite contraction in laboratories. While Sahadath et al. developed a database for radiation shielding parameters of concrete containing magnetite and ilmenite [33], Oto and Gür obtained and theoretically calculated the linear attenuation coefficients of concrete specimens containing magnetite using the WinXCOM computer code [74]. Using experimental data, MCNP and XCOM software, Sayyed et al., investigated the gamma radiation shielding performances of concrete containing sepiolite and boron carbide ($B_{4}$C) [28], and Davraz et al. investigated concrete specimens made with $B_{2}O_{3}$ additive, boron modified active belite, and portland cement for neutron shielding purposes [75]. Although silico computation based on XCOM or Montecarlo is cost-effective, artificial intelligence technologies are also a hot topic in academic research because they can perform optimization operations independently of datasets to forecast new concrete powder mixtures that are cost-effective in shielding from various types of radiations or provide categorization and regression results by operating their algorithms on datasets. Additionally, there are a number of techniques in the literature that employ artificial neural networks (ANN) and other kinds of artificial intelligence to forecast concrete’s mass attenuation coefficient and radiation shielding properties. For example, Medhat used the ANN technique to predict the mass attenuation coefficient in various materials and compared the results with the data he theoretically obtained using the XCOM program with the results from his experimental measurements [56]. Gencel, meanwhile, used Monte Carlo (MCNP) and artificial neural network (ANN) to predict the mass attenuation coefficient of the harzburgite mineral [51]. Additionally, Yadollahi et al. examined the radiation shielding capabilities of concretes using artificial neural network (ANN) [55] and utilized the Taguchi technique to determine the ideal mix of concretes including lead-slag aggregate. Using various proportions of barite and regular aggregate, Klnçarslan made concrete and experimentally determined the radiation attenuation coefficient of such concretes [52]. Given that we implemented well-known ML methods in an effort to advance the discipline. As can be seen, several theoretical and practical experiments have been conducted to develop a concrete type that is efficient at radiation shielding. Unlike the literature mentioned above, we implemented and evaluated eight distinct ML models. In general, limited datasets are used to train and evaluate AI models on this subject. We employed a method as shown in Section 3.10 while dealing with a dataset of 14,758 values, which may be regarded as substandard and ML could not perform effectively. We used XCOM to simulate the LAC, and then we fed the same dataset into forecasting ML models with the goal of predicting a linear attenuation coefficient that was as similar to the XCOM-simulated one as possible. The benefit of this method increases when reliable algorithms are identified and utilized to forecast new samples in order to simplify the mixing process of new composite with enhanced shielded features. Furthermore, ML models may be trained in a computer lab in a matter of minutes and on a small dataset, in contrast to the more time- and resource-intensive deep learning. In this sense, our research represents a development of the existing literature on using ML for the prediction of new concrete mixtures with enhanced shielding properties. As is customary for this topic, the differences between ML and XCOM’s forecasted and simulated outcomes are examined in terms of regression, statistical analysis, and the effectiveness of ML in terms of MAE, RMSE, and R2score.

## 6. Conclusions

There is little question that radiation protection research will keep growing as the usage of radiation and its effects on human life expand with technological advancements. Concrete is one of the materials used to guard against radiation. In this work, magnetite powder, one of the unique forms of concrete created for specific purposes and applications, was added to experimental data of radiation attenuation coefficients, and the shielding properties of various combinations were predicted using eight ML algorithms. A comprehensive and reliable database of the radiation shielding characteristics of concrete mixes made up of mineral powder particles and heavy material mixtures at various water/density ratios were produced using the XCOM tool on a handwritten dataset. Then, using the same dataset, we trained and evaluated eight well-known ML regressors with the goal of forecasting a LAC that was roughly similar to the one generated by XCOM. According to the results, our built HELM network outperformed different machine learning algorithms, such as SVM, decision tree, polynomial regression, random forest, MLP, CNN, and ELM, with the greatest correlation score (r = 0.999) between HELM and the XCOM LAC. In this paper, a brand-new Linear Attenuation Coefficient (LAC) forecasting method based on hierarchical extreme machine learning was proposed. To the best of our knowledge this is the first research to use the HELM for LAC prediction. HELMs may also be used to more effectively, consistently, and accurately forecast the quality characteristics of RSC. The potential benefits might be an essential design guide for radiation shielding engineering. In addition, instead of cumbersome laboratory tests that consume a significant amount of raw materials, a more ecologically friendly mix design for cement concrete might be developed. Last but not least, the HELM-based technique may be applied in other industrial chains, such as cement production processes, for calculating radiation shielding capacities of materials. This belief is prompted by the encouraging results in this work.

## Figures and Tables

**Table 1:** Descriptive overview for the employed experimental database.

Constituent	Weight (g/cm${}^{3}$)				
	$W_{1}$	$W_{2}$	$W_{3}$	$W_{4}$	$W_{5}$
CaO	0.390	0.152	0.187	0.218	0.247
MgO	0.390	0.284	0.188	0.099	0.0187
NaO	0.006	0.004	0.003	0.002	0
$K_{2}0$	0.011	0.008	0.005	0.003	0.0002
$Fe_{2}O_{3}$	0.008	0.089	0.163	0.230	0.292
$P_{2}O_{5}$	0	0.0005	0.001	0.001	0.001
$CO_{2}$	0.082	0.060	0.040	0.022	0.005
$SiO_{2}$	0.249	0.258	0.267	0.274	0.282
$Al_{2}O_{3}$	0.039	0.034	0.029	0.025	0.021
$SiO_{2}$	0.005	0.004	0.004	0.004	0.003
$TiO_{2}$	0	0.0000916	0.000175109	0.0002515420	000321765
BaO	0	0.0000458	0.00008755	0.000125771	0.000160883
$Cr_{2}O_{3}$	0	0.002404626	0.004596616	0.00660298	0.008446339
$Na_{2}O$	0	0.000480925	0.000919323	0.001320596	0.001689268
$SO_{3}$	0	0.000549629	0.001050655	0.001509252	0.001930592
$V_{2}O_{5}$	0	0.00001374	0.000026266	0.000037731	0.000048265
ZnO	0	0.002184774	0.004176354	0.005999279	0.007674102
Magnetite%	0	0.25	0.5	0.75	1
Density	2.44	2.55	2.67	2.78	2.9
$H_{2}O$	0.0787	0.0751	0.0717	0.068	0.065

**Table 2:** MAE, RMSE and R2score performance of the proposed regressors, evaluated on test sets.

	MAE	RMSE	R2score
SVM	0.441 ± 0.356	0.457 ± 0.362	0.286 ± 0.315
Decision Tree	0.457 ± 0.314	0.543 ± 0.294	0.437 ± 0.293
Polynomial Regression	0.328 ± 0.301	0.369 ± 0.322	0.369 ± 0.321
Random Forest	0.553 ± 0.303	0.487 ± 0.305	0.439 ± 0.305
MLP	0.359 ± 0.374	0.348 ± 0.393	0.667 ± 0.277
CNN	0.574 ± 0.387	0.422 ± 0.308	0.530 ± 0.334
ELM	0.442 ± 0.356	0.408 ± 0.361	0.516 ± 0.296
HELM	0.360 ± 0.304	0.158 ± 0.302	0.753 ± 0.292
